# Transforming Perspectives Through Virtual Exchange: A US-Egypt Partnership Part 2

**DOI:** 10.3389/fpubh.2022.880638

**Published:** 2022-05-23

**Authors:** Sarah L. Collins, Savanah Mueller, Elizabeth A. Wood, Nichole E. Stetten

**Affiliations:** ^1^College of Public Health and Health Professions, University of Florida, Gainesville, FL, United States; ^2^Department of Environmental & Global Health, College of Public Health and Health Professions, University of Florida, Gainesville, FL, United States; ^3^Department of Occupational Therapy, College of Public Health and Health Professions, University of Florida, Gainesville, FL, United States

**Keywords:** virtual exchange, global learning, transformative learning, collaborative online international learning, qualitative

## Abstract

Collaborative online international learning programs, such as virtual exchange, that utilize telecollaborative activities have been integrated into more classrooms within the higher education setting. These programs provide students exposure to international cultures, perspectives, and ideas is no longer considered “value added”, but a prerequisite to entering many workforces. These programmatic objectives compliment Mezirow's Transformative Learning Theory, that substantiates two major elements of transformative learning are critical reflection and dialectical discourse. This study presents the second half of a qualitative inquiry into the prominent themes that arose during a virtual exchange that was conducted in March 2021 between students in the United States (US) enrolled in a global public health course and Egyptian microbiology students. This study sought to expand upon the Transformative Learning Theory through inductive analysis procedures to offer a modernized adaptation of the theoretical framework within international learning environments. Student responses enrolled in an undergraduate global public health course were collected and analyzed by two coders using inductive/open coding to identify salient codes. These codes were then summarized into categories and subsequently defined. Resulting themes include Connectedness, Openness, Acquisition of Knowledge and Skills, Communication, Cultural Identity, Anticipation of Options for New Roles, Relationships, and Actions, and Absence of Change. Several themes have corresponding categories and subcategories. Adult learning environments such as the modern college classroom have changed with the introduction and reliance upon online learning domains, as well as the diversification of higher education student demographics, accentuating the need to inductively analyze student learning processes and outcomes. In doing so, our findings provide a modernized adaptation of the Transformative Learning Theory that allows for adult learning theorists, researchers, and scholars to integrate tenets of transformative learning more appropriately. As such, this provides an opportunity for educators to coalesce the identified mechanisms (e.g., openness, cultural background, anticipation of roles and relationships) to bolster student's willingness and ability to engage in transformative critical reflections. By capitalizing on students' innate characteristics, such as open-mindedness predispositions and cultural background, educators are able to augment transformative learning strategies through tailored assignments and course activities.

## Introduction

Collaborative online international learning, or COIL, has been integrated into more classrooms in higher education to advance areas around intercultural competence as well as to improve dialogue and digital literacy skills through telecollaboration (among other methods). COIL, often referred to by other monikers such as virtual exchange, is a strategy employed initially by foreign language instructors and in business classes to bolster international collaborations (1–4). Within the contexts of this study, we chose the broader term Virtual Exchange (VE) to describe the series of telecollaborative activities that were implemented in the classroom. To provide students exposure to international cultures, perspectives, and ideas is no longer considered “value added”, but a prerequisite to entering many workforces (5–7). Implementing elements of COIL can be done within the classroom or through other mechanisms, such as student organizations or as a program requirement. Internationalization, especially during the time of the COVID-19 pandemic, has reinforced the importance of integrating intercultural competence within courses to prepare students for diverse societies (6).

Intercultural competence, a common principle within VE research, has been defined in several ways but most definitions include four constructs that are measured and/or included within implementation (6, 8–11). These constructs (attitudes, knowledge, skills, and behaviors) contribute to the students' overall understanding of self, having the agency to want to discover or explore the unknown, and similar to concepts within cosmopolitanism (12), going beyond one's own perspective and self (13–15). Facilitating factors within intercultural competence include a more immersive experience when learning about course content than many traditional teaching formats (6, 7, 16). Interacting with international peers directly, through a virtual modality, students create the opportunity to better understand complex dynamics through their own lived experiences (17). While most pedagogical approaches to increasing intercultural competence include mobility, which may include study abroad, financial constraints and time often limit students from engaging in these activities (6, 16, 18). Moreover, with less than ten percent of undergraduate students in the United States participating in study abroad, it does not appear to be the most pragmatic approach to addressing intercultural competence (6, 19). While VE can be applied to most if not all disciplines, the students' lack of awareness of their international peers' local language can have a deleterious impact on overall student learning outcomes through breakdowns in communications, misunderstanding expectations of presented tasks, and differing interpretations of how to problem solve (16, 20–23). Without offering students the opportunity to reflect on how diverse perspectives catalyze varying interpretations, there can be no reconciliation (24–27). For example, resolving issues around time differences is common among VE programs and does not necessarily create resentment or negative feelings. This is especially important as we review and make recommendations for Mezirow's Transformative Learning Theory (TLT) (28) around students feeling shame, regret, or other negatively charged feelings within the VE.

### Transformative Learning Theory

The TLT is a critical adult learning theory grounded in the belief that “learning is understood as the process of using prior interpretation to construe a new or revised interpretation of the mean of one's experience in order to guide future action” (29). Mezirow (29) further elaborates individuals make sense of ideas and communicate through their frame of reference, which is composed of meaning perspectives and meaning schemes. Meaning perspectives are considered to be broad and orienting predispositions whereas the meaning scheme is a group of beliefs, feelings, attitudes, and judgements that place meaning or shape a specific interpretation. Within the TLT lens, learning occurs by elaborating on existing meaning schemes, learning new meaning schemes, or transforming meaning schemes (29). The mechanisms by which these occur are through either instrumental or communicative learning. Instrumental learning depicts the idea of seeing a cause and effective relationship, whereas communicative learning is the process of deciphering what the intended meaning or feeling others are expressing when others communicate with us.

**Figure 1:**
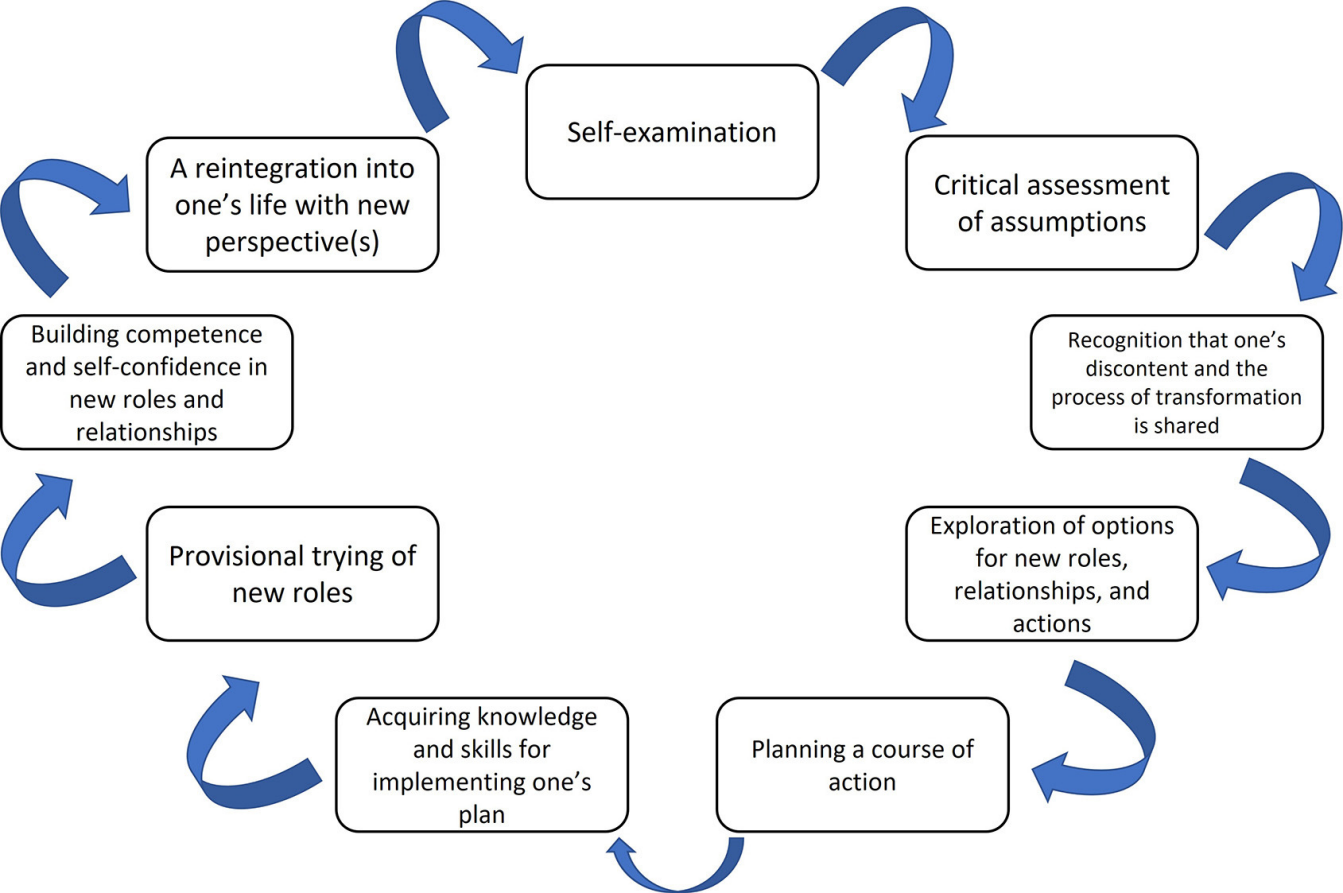
Interpretation of Mezirow's transformative learning theory.

The TLT is thought to occur in a stage-base cyclical process, originally constructed from Mezirow and Marsick's research on re-entry of women into college programs (30, 31). The most recent revision of the conceptualization of the TLT was published in 2006 (28). However, throughout each revision of the TLT, it has maintained the stage-based cyclical process and has been employed within modern research efforts such as Sharpe's work on enabling behavior change for adaptation and resilience to disaster threats using the TLT (32). The visual depiction of Mezirow's (28) TLT can be found in Figure 1.

Mezirow's recent work substantiates that the two major elements of transformative learning are critical reflection and dialectical discourse (28). The VE described in this study sought to provide opportunities to both critically reflect and engage in dialectical discourse with various international parties. This study is the second half of a qualitative inquiry into the prominent themes that arose during a VE that was conducted in March 2021 between students in the United States (US) enrolled in a global public health course and Egyptian students enrolled in a microbiology course. The prequel to this study (33) presents the manner in which students validated Mezirow's TLT (28) through deductive classification in the existing stages. In contrast, this study sought to expand upon the TLT (28) through inductive analysis procedures. Evidence suggests that adult learning environments such as the modern college classroom have changed with the introduction and reliance upon online learning domains (34, 35), as well as the diversification of higher education student demographics (36, 37), thus accentuating the need to inductively analyze student learning processes and outcomes that allow for salient themes that provide an expansion of learning theories that were developed in a different learning era.

## Methods

### Data Collection

University of Florida (UF) students enrolled in PHC3440: Global Public Health participated in a 5-week Virtual Exchange (VE) with students from Ain Shams University (ASU) in Cairo, Egypt in Spring 2021. The VE experience was accompanied by a series of reflection-based assignments, collective which were referred to as the Global Learning Experience (GLE) within the course. More specifically, students responded to an adapted version of the State University of New York's COIL Stevens Initiative assessment (38), as well as a comprehensive Individual Analysis Paper (IAP), resulting in four time points of data collection. A more comprehensive overview of the operationalization of the GLE (33), as well as the programmatic structure (Appendix A) have been described elsewhere. A total of 108 UF students engaged in reflection-based assignments throughout the GLE.

Upon semester completion, the course instructor exported and de-identified student submissions, providing each student with a unique code to indicate their reported sex, race/ethnicity and a randomly selected number. The research team stratified students according to reported sex and found that only 11 students reported being male. In an effort to ensure male participants were included in analysis, the research team conducted a 2:1 match sampling procedure based on each male's reported race/ethnicity. Therefore, reportedly female respondents were stratified according to their reported race/ethnicity and randomly selected until the 2:1 quota was met. This was conducted to ensure a representative and appropriately sized sample. Once selected, student responses to the four reflection-based assignments were collected. Students were excluded from the final sample if they did not complete all assignments or whose responses were severely lacking in addressing the assignment prompt. This resulted in the final sample of 28 students.

The student responses were organized by each time point and responses were collated according to the assessment item. For example, the first reflection-based assignment had four items. All responses to item one were collated on one document. Student responses were labeled corresponding to their unique code. More details regarding the data collection and sampling approach can be found in Part 1 (33). The UF Institutional Review Board approved this study (IRB#: IRB202003293).

### Data Analysis

Student responses were analyzed using inductive and directed content approaches. The researchers used Mezirow's TLT as the guiding theoretical framework to conduct directed content analysis, which specifically seeks to expand upon or validate an existing framework (39). The results of the directed content analysis are published in Part 1 (33), with this paper focusing solely on inductive results. For codes that did not fit into the predetermined themes (constructs within Mezirow's TLT), inductive/open coding was used to identify salient codes. These codes were then summarized into categories and subsequently defined (39). Some themes contain categories and subcategories that provide a more specific phenomenon.

Two coders first independently conducted analysis procedures, hand coding line-by-line. Upon the conclusion of open coding, like-codes were grouped and labeled to develop a new category (40). Negotiations between the two coders were performed after each data set from the different time points was completed. During each negotiation, the researchers redefined the operational definitions based upon the findings and re-coded, line-by-line, until they reached complete agreement. Following the completion of the IAP data analysis negotiation, the researchers revisited all data to ensure continued fit to the finalized category operational definitions.

Content analysis procedures not only provide a systematic approach to describing phenomena, but also a means to quantifying such phenomena (41). The frequencies of each theme were calculated upon the conclusion of data analysis. Themes were only counted once per student response per item, despite the frequency of presentation. Some student responses contain more than one theme.

## Results

A total of 28 students were selected for the sample to represent the 108 undergraduate students partaking in the GLE program. These students were majority female and comprised a diverse cultural and ethnic group including White, Non-Hispanic, White-Hispanic, Black, Asian, and two or more races. The final themes, categories and subcategories and their respective operational definitions can be found in Table 1. Frequencies are presented in Table 2. Results are presented in order of prevalence.

**Table 1:** Finalized themes, categories, and sub-categories.

**Themes**	**Categories (If applicable)**	**Sub-categories (If applicable)**	**Operational definitions**
Connectedness			Contextualizing the depth and breadth of personal and professional linkages across people and populations and the international flow of ideas.
	Relatedness		Situating one's self and country/culture in relation to international partners and objectively identifying and examining similarities and differences.
	Friendship		Informal social relationships in which one engages in subjective, “fun”, pleasant interactions with others.
Openness			An objective expression of one's willingness and/or present opportunity to grow knowledge, understanding, and/or skills based upon this collaborative online experience. In addition, a perceived willingness from international partners to share and engage in open conversations.
	Global & health perspective		A specific willingness or opportunity to develop or expand worldly and Egyptian cultural knowledge, understanding, and skills (i.e., values, beliefs, priorities), but specifically not related to public health. In addition, a perceived willingness of Egyptian students to expand or develop American perspectives.
	Public health perspective		A specific willingness or opportunity to develop and expand an awareness on Egyptian and global public health. In addition, a perceived willingness of Egyptian students to expand or develop American public health perspectives.
Acquisition of knowledge and skills			A noted [passive] statement of one's engagement in the ongoing process of procuring and developing knowledge and skills through international relationships.
	Acquire and institutionalize cultural knowledge and skills		A noted [passive] statement of one's engagement in the ongoing process of procuring and developing both intercultural and intracultural knowledge and skills. This is a foundational step in developing cultural competence.
	Acquire and institutionalize public health knowledge and skills		A noted [passive] statement of one's engagement in the ongoing process of procuring and developing both intercultural and intracultural public health knowledge and skills.
Communication			Encompasses any explicit mention of one's ability to dialogue and converse with partners to achieve PHC3440 objectives.
	Difficulties in communication		A mention of barriers that impeded communication efforts between international partners.
		Challenges in virtual exchange communication channels	A specific description of logistical difficulties such as internet connection, online communication platforms, time, etc. that hindered one's ability to communicate effectively with partners, potentially causing discourse.
		Challenges in communication due to language barrier	A specific description of language barriers including formality and comprehensibility of terms, phrases, etc. that hindered effective communication with partners, potentially causing discourse.
	Successful communication		A specific mention of positive experiences and mechanisms to dialogue with partners.
Cultural identity			A portion of one's sense of self that derives from their racial and ethnic origins, practiced traditions, and/or values and beliefs.
	Multicultural		Self-identified cultural background originating from more than one racial and ethnic origins, practiced traditions, and/or values and beliefs that influences one's sense of being.
		Manifestation of multicultural identity	A personal description of one's values, traditions, and identity that depicts a differential boundary between two cultural backgrounds and their interactions with one another.
	Monocultural		Self-identified cultural background originating from one racial and ethnic origin, practiced tradition, and/or values and beliefs that influence one's sense of being.
	Manifestation of cross- cultural interactions		Expressed understanding of, feelings toward, or appreciation for international persons based upon the participants cultural background and identity.
Anticipation of options for new roles, relationships, and actions			Participant preemptively contemplates opportunities to integrate and apply new [assumptions] *via* personal identity, interactions, and personal behaviors based on PHC3440 course structure.
Absence of change			An explicit statement from the participant claiming that they believe they will not have (pre) OR have had little to no shift/change in perspective or assumptions (mid-, post-, IAP).

**Table 2:** Frequency of themes, categories, and sub-categories.

**Themes**	**Categories (If applicable)**	**Sub-categories (If applicable)**	**Data collection round**				
			**Pre-VE**	**Mid-VE**	**Post-VE**	**IAP**	**Total**
Connectedness			16	5	10	12	**43**
	Relatedness		15	40	37	24	**116**
	Friendship		8	6	1	4	**19**
Openness			21	24	14	4	**73**
	Global & health perspective		31	8	13	6	**58**
	Public health perspective		27	0	7	2	**36**
Acquisition of knowledge and skills			3	7	17	8	**35**
	Acquire and institutionalize cultural knowledge and skills		1	11	5	12	**29**
	Acquire and institutionalize public health knowledge and skills		0	14	24	24	**62**
Communication			0	0	0	0	**0**
	Difficulties in communication		0	2	7	3	**12**
		Challenges in virtual exchange communication channels	0	6	22	25	**53**
		Challenges in communication due to language barrier	0	2	0	5	**7**
	Successful communication		0	17	13	28	**58**
Cultural identity			1	0	7	16	**24**
	Multicultural		18	6	3	6	**33**
		Manifestation of multicultural identity	6	1	3	12	**22**
	Monocultural		7	7	4	6	**24**
	Manifestation of cross- cultural interactions		5	11	7	6	**29**
Anticipation of options for new roles, relationships, and actions			16	3	1	1	**21**
Absence of change			3	6	6	5	**20**

### Connectedness

Connectedness was divided into two categories: (1) Relatedness and (2) Friendship. Relatedness was the most frequently demonstrated construct throughout the VE. This theme and its categories were equitably distributed across all four timepoints. Characterized by students' appreciation toward the exchange of information, students described “how important different perspectives are when addressing global public health issues. It is completely unhelpful to study global public health issues in a vacuum, as there will always be another perspective that adds valuable information to creating a solution.” Students shared a mutual understanding with their Egyptian peers as demonstrated here where a student writes, “It seems that there is a sub-category for people that come from countries outside of the US and it allows me to feel comfortable being able to interact with people who also are not originally from the US.”

#### Relatedness

In most cases, students found themselves more similar to their Egyptian counterparts than expected, “Our similarities are much greater than our differences.” As the exchange was centered on the COVID-19 pandemic, students would compare their governments' COVID-19 responses, “I did learn that our governments dealt with COVID-19 in a similar fashion.” In addition to the government response, students found similarities in personal circumstances as well, “They have kind of the same hopes/fears about the pandemic and that our responses weren't so different. An example of this is how their university shifted its curriculum, however, it was different in that they had projects but still similar in that all their classes went online.” Students went further than finding a common background to enhance understanding, but similar cultural circumstances lent themselves to creating more substantial relationships and conversation. One student said, “Most times when I come across someone who is not originally from the United States, I find that I am able to relate to them more easily and have a better time connecting because immigrant ideals tend to be pretty similar in certain aspects.” Identification and acknowledgment of this shared history or experience was a key component in Relatedness.

#### Friendship

Students admitted to hoping that they would “make some friends” during the exchange. Admittedly, some students were disappointed at the end of the exchange that they did not get to experience more of these interactions. Students appreciated informal interactions examining each others' social lives such as “When I shared that I am a professional musician who has a YouTube channel, they were quick to check out my content and were so loving and encouraging.” This also demonstrated the receptiveness and active engagement from both parties.

### Openness

Openness followed Relatedness in frequency. This theme was divided into two categories: openness to (1) global and cultural perspective and (2) public health perspective. Prior to the exchange, students would express what they hoped to gain such as, “I hope to meet new people and be able to learn about their lives...I want [to] be a little less ignorant walking away from this experience.” Some students' personal experiences served as a motivator of remaining open and accepting of their peers. For example, “I do not like when people make assumptions about me, so I keep an open mind when talking to people from a different background than me.” During the latter half of the VE, students recognized the value of openness as a result of their interactions. One student admitted, “This course has taught me the importance of being open-minded and willing to participate.”

#### Global and Cultural Perspective

This Openness category depicts a specific willingness or opportunity to develop or expand worldly and Egyptian cultural knowledge, understanding, and skills. This was most frequently demonstrated prior to the VE. As one student said, “Overall, what I want from this virtual exchange experience is to take a deep dive into another culture.”

#### Public Health Perspective

The second category of Openness encapsulated a willingness to develop or expand an awareness of Egyptian and global public health. Students anticipated the exchange enhancing their public health knowledge and ability to address public health needs with frequency piquing during the pre-VE timepoint. For example, “The interactions I have with these students will impact my learning in this course as it will place a greater emphasis on understanding how culture can impact public health interventions” and “I would want to further explore public health responses in other countries to see what they do better and worse.”

### Acquisition of Knowledge and Skills

Acquisition of Knowledge and Skills was divided into two categories relating to Cultural Knowledge and Skills and Public Health Knowledge and Skills. Students recognized different perspectives (knowledge) which, “created a new dimension to consider when creating policy…there are no rights or wrongs, simply different but equally relevant approaches to the same problem.” Any time students admitted to learning something new *via* a passive interaction, thereby not specifically asking questions or researching, this was defined as the acquisition of knowledge and skills.

#### Acquire and Institutionalize Cultural Knowledge and Skills

The acquisition of knowledge and skills in relation to institutionalizing cultural knowledge was a noted [passive] statement of one's engagement in the ongoing process of procuring and developing both intercultural and intracultural knowledge and skills. In these instances, students explicitly described passive “learn[ing] a lot about their culture, values, and their country as a whole.”

#### Acquire and Institutionalize Public Health Knowledge and Skills

This category illustrates one's engagement in the ongoing process of procuring and developing both intercultural and intracultural public health knowledge and skills, and followed Openness in frequency. Students would relate what they learned through conversing with their peers to their curriculum and current understanding of public health. For example, one student said, “Working with students in another country has helped me to get a better insight into the quality and status of public health in a country I was previously unfamiliar with. It has also allowed me to view the systems we have in place in the United States with a more critical eye and with more appreciation simultaneously.” Many students echoed this sentiment and further added that learning from the textbook is not sufficient when they could gain “inside access to another country's public health issues from those who experience it themselves.”

### Communication

Regarding the VE, students were asked about their ability to communicate with their Egyptian counterparts. Different facets of communication were divided into categories including difficulties in communication and successful communication.

#### Difficulties in Communication

Difficulties in communication included a mention of barriers that impeded communication efforts between international partners. Often, this would present as social anxieties. A few students mentioned worry in conversing such as, “I think this experience was stressful when I was first encountering the international students. I was very fearful that I would unintentionally offend them or lack commonality.” Frequently, challenges revolved around communication channels such as, “The time difference between Florida and Cairo was definitely a barrier in this project.” These channel-communication barriers also included the unreliability of communication apps and learning management systems (e.g, WhatsApp, Canvas, and Zoom. Concerns over Egyptian students' knowledge of programs like Google Docs or Google Slides were expressed, as well as issues pertaining to video conference connectivity.

Another challenge was language and English dialect. For example, “The Egyptian students were very advanced in their English, but there was still a barrier at times with them fully understanding complicated phrases and words.” It is notable that while these challenges were evident, most students followed up these statements with an ability to adapt and overcome the issue.

#### Successful Communication

Contrary to difficulties in communication and, noted almost as frequently, was successful communication. This was defined as a specific mention of positive experiences and mechanisms to dialogue with partners. Many students enjoyed the ability to converse with various apps such as Whatsapp. Both Egyptian and American students were receptive to the exchange platform, with many American students providing feedback such as “My partners from Egypt have been very amiable and easy to communicate with.”

### Cultural Identity

Students were asked to define their own cultural identity in response to the prompts. These responses were categorized as multicultural, or monocultural, and manifestation of cross-cultural interactions. Being aware of their own cultural background and experiences “really does impact the way they view the world.” For example, “there are a lot of differences between my parents' views of the world and a lot of American-born people. I really like this because I think that these differences allow me to create a more complete view of the world rather than a one-sided one.”

#### Multicultural

Student responses were coded as multicultural when they self-identified their cultural background as originating from more than one ethnicity or race that influences one's values, traditions, and sense of being. Examples included American and Korean and Pakistani-American. One student identified them self as diverse, “As a biracial, first generation American, my cultural background is diverse.” Students also elaborated on how these two or more identities would manifest, whether in tandem or contrasting, resulting in the sub-category, manifestation of multicultural identity. For example, “I have also struggled with my identity as a Latina in America because of the inability to entirely fit in either American or Latinx spaces.”

####  Monocultural

Students who identified as monocultural, self-identified their cultural background as originating from one ethnicity or race that influences one's values, traditions, and sense of being. Numerous students classified themselves as American. For example, “Going into this experience, I would describe my cultural background as very much American.” But monocultural was not limited to this and another student added, “My cultural background is of Haitian descent.”

####  Manifestation of Cross-Cultural Interactions

In addition to cultural background self-identification, students examined the influence of their cultural background on their interactions through expressed understanding of, feelings toward, identify with, or appreciation for international partners based upon the participants cultural background and identity. Individuals described being more sensitive to cultural nuances due to their acknowledgment of their own cultural background. For example, “I think my background influenced my understanding of how to collaborate with students abroad by helping me to not put people into categories.” Students explained that their own cultural background facilitated an environment of respect, understanding, and openness. One student explained, “I respect people's culture just as I want others to respect my culture…my background has influenced me to think a certain way but respect people's opinion other than my own. This allows me to understand why people do a certain thing even when I do not agree with it.” This discussion demonstrated some internal reflection from students, fostering greater relationships with their peers.

### Anticipation of Options for New Roles, Relationships, and Actions

Prior to the VE, and during limited occurrences throughout, students would anticipate their options for new roles, relationships, and actions. The expression of this theme often involved the ability to apply course content or information gleaned from the VE in the future. Before the exchange, students described how they would use new information, “I want to take this perspective into my future career as a physician and treat my future patients from other cultures with personalized care in light of their cultural preferences, ensuring their sense of comfortability.”

### Absence of Change

This theme occurred throughout all four timepoints, although rarely. Students would explicitly mention that they did not anticipate any change in attitudes nor did not recognize any change after the fact. For example, “I do not think I experienced any strong changes in how I view the world.” Many students who noted this theme, did follow this statement with a “However,” clause indicating some change, although they may not have explicitly identified or acknowledged it.

## Discussion

Global Learning Experience (GLE) programs offer telecollaborative, immersive activities that provide students with exposure to international cultures, which has now become a prerequisite for many careers (5–7). One of the instructional goals within GLE is to increase intercultural competence, which ultimately seeks to contribute to a student's understanding of self, increase their sense of agency to discourse or explore foreign ideas or principles, and transcending one's own perspective and self (13–15). These GLE aims complement Mezirow's TLT, which specifically posits that learning occurs by elaborating on, learning new, or transforming existing attitudes, beliefs, and understandings of a phenomenon (29). This study sought to implement a GLE program in an undergraduate global public health course grounded in the TLT, using student assignments as a means to validate, expand, or offer a critique of the current TLT through qualitative, inductive methodologies.

**Figure 2:**
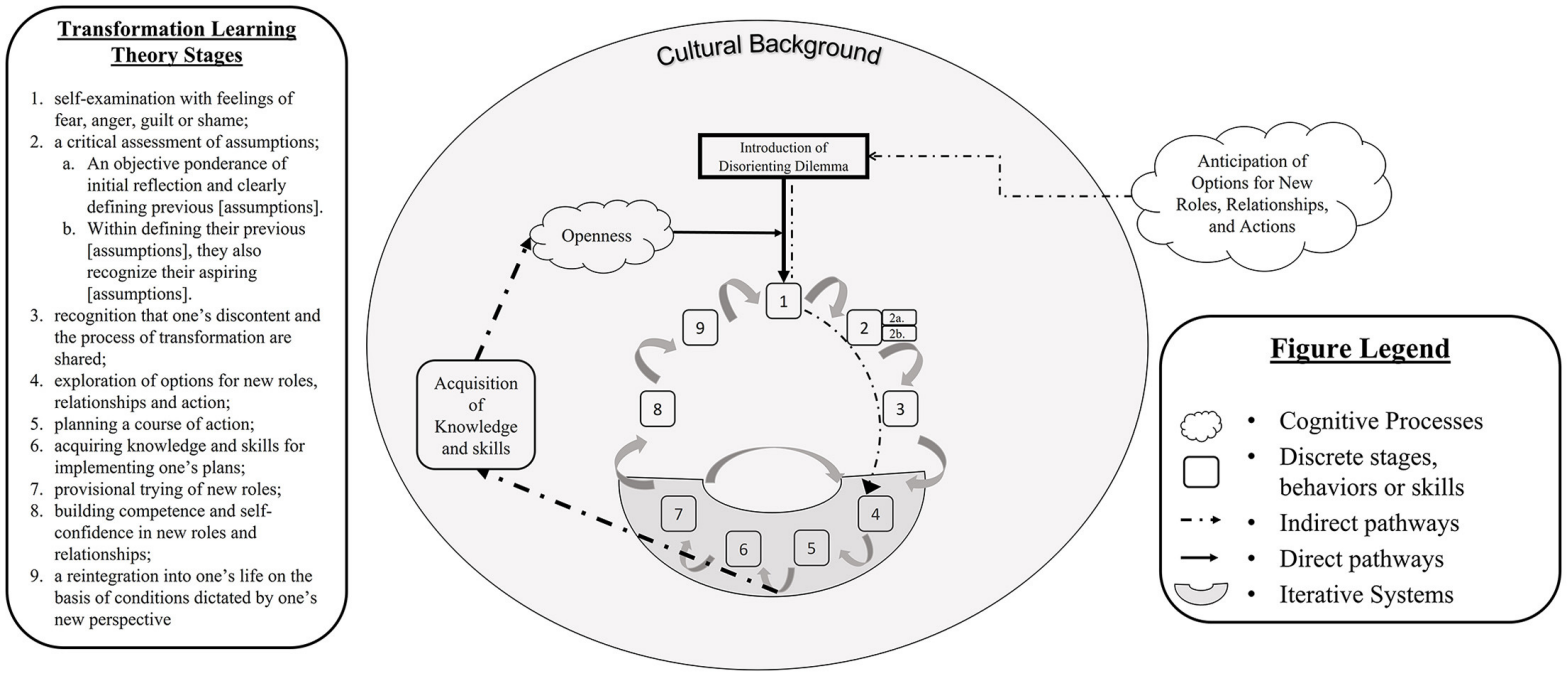
Modernized adaption of transformative learning theory.

Part 1 of this study demonstrates the manner in which students' perspective-taking, self-awareness, and critical reflections coincided with Mezirow's TLT (28). In contrast, this study sought to explore how students' learning processes deviated from Mezirow's TLT. It is essential to openly analyze student learning experiences, as the original TLT was developed during a vastly different era - when women were just starting to enter collegiate domains (30). In contrast, today's higher education student make-up continues to diversify (36, 37), while learning domains and platforms transform as the digital age continues to evolve (34, 35). Therefore, a revised interpretation of the TLT conceptual framework is provided within Figure 2 that integrates our inductive findings.

Our findings demonstrate that the TLT may be vulnerable to a student's preemptive beliefs about the value of or excitement toward a disorienting dilemma. For example, “Openness” was the second most prominent theme and we found that students who were more open in the pre-GLE assessment were more likely to enter into the cycle of TLT. However, those that reported they believed no change would occur often cited an experience of an absence of change, thus demonstrating a potential influence of their preemptive mindset. This is not surprising considering Mezirow's (42) claim that one's critical self-reflection is more likely to be emancipatory when met with ideal learning conditions, one being openness to alternative perspectives. However, the prevalence of students' openness toward this learning experience may illustrate that the current TLT underestimates the influence of one's origin mindset. Therefore, one's openness may be more appropriately considered as a gatekeeping mechanism that serves as an enabler or inhibitor to one engaging in critical self-reflection rather than strictly as an ideal condition (42). We found that upon the conclusion of the GLE students reported having attained openness as a skill. Similarly, others have found that exposure to collaborative learning opportunities increases one's openness to diversity (43). This increase in openness to diversity likely perpetuates one's excitement for more opportunities/disorienting dilemmas that present alternative perspectives that challenge an individual's presuppositions.

Within our findings, that value of “Connectedness” was emphasized by one of its subthemes, “Relatedness,” being the most frequent theme identified within student responses. This coincides with existing literature that demonstrates global connectedness can be enhanced through curricular opportunities that engage in cross-cultural dialogue and contexts within higher education settings (44). Students reported a high degree of relatedness, indicating that they were more similar to their international counterparts than originally thought. This is likely exacerbated within a Euro-American cultural context that situates otherness as a central tenet within our individualized identities (45, 46). Combatting this principle of otherness through the GLE allows for students to identify similarities rather than emphasizing differences, thus mechanizing critical reflection and rebuilding meaning schemes for one to operate within in future educational and occupational endeavors. However, not all cases of relatedness were presented in terms of a surprise. Several students indicated feeling closer to the Egyptian students than their American peers based on their cultural background. Existing literature cites that acculturation processes often accompany international students (47), non-traditional students such as veterans (48), and students with immigrant background (49). Consequently, students with reported multi-cultural backgrounds may have already endured acculturation processes that allow them to relate more closely with international partners than their American peers. These acculturation processes often require that students engage in self-identification to assess their sense of belongingness, centrality to the major culture, and traditions that may coincide or deviate from major culture norms (50). This can ultimately be thought to prime students to have a higher degree of cultural awareness than students who have had the privilege of innately belonging to the majority culture within higher education. Therefore, cultural background should be considered when assessing one's degree of transformational learning.

“Openness” and “Connectedness” presented new mechanisms which facilitated transformational learning. In contrast, “Acquisition of knowledge and skills” and “Anticipation of Options for New Roles, Relationships, and Actions” can be seen as accompanying steps within the TLT conceptual framework rather than as separate or substantial pathways for transformative learning. By assessing students before the onset of the disorienting dilemma (VE), students preemptively contemplated options for new roles, relationships, and behaviors that were eventually revisited within the traditional TLT stage four, “Exploration of options for new roles, relationships and action” (33). Therefore, allowing students to contemplate potential uses for altering their preconceived notions may encourage openness and increase the likelihood for these new options to be realized. In addition to these new mechanisms, we identified that the traditional TLT steps four through seven (33), may offer a separate iterative process within the larger transformational learning cycle. Students showed that after provisionally trying new roles, they often revisited exploring new options if something did not work out. This is intuitive as the “provisional” trying of new roles suggests a troubleshooting mechanism. However, within this iterative process, students reported acquiring and institutionalizing new skills and behaviors in a more implicit manner. These implicit indications were categorized under the “Acquisition of knowledge and skills” because these skills may be unrelated to the transformation of their previous assumptions, but rather allow for skills such as open-mindedness, that will benefit students in future opportunities of transformative learning.

Despite an overwhelming number of students acknowledging the benefits of VE, there were frustrations expressed by students around communication Many students discussed having international partners who were attentive and interacted regularly, while, other students described situations where their partners were unresponsive, causing them stress and/or anxiety due to upcoming assignments. Guth and Helm (38) address issues around communication through stating explicit meeting times that are negotiated beforehand and working with students on larger communication dynamics. Contrary to literature recommending more training in online communication (51), due to the COVID-19 pandemic, students internationally were sprung into online settings that afforded them the ability to improve their digital literacy skills. In fact, by offering the students more than one online modality to communicate, students felt more in control of their assignments and activities within the module, which was emphasized in previous literature (52, 53). As most studies around VE have underscored already, the use of online technology in promoting collaborative learning is essential in its success (54, 55). Technology-supported environments for social interactions and experiential learning can foster reimagined pedagogical approaches to teaching course content (55). This is especially relevant as the COVID-19 pandemic has catalyzed more momentum for online learning, cultivating digital skills, and addressing social equity issues around international travel (55, 56).

This study allows for scholars to integrate a modernized version of Mezirow's TLT (28), that accounts for the influence of the digital age on learning environments, as well as the diversification of students in higher education. As such, this provides an opportunity for educators to coalesce the identified mechanisms (e.g., openness, cultural background, anticipation of roles and relationships) to bolster student's willingness and ability to engage in transformative critical reflections. By capitalizing on students' innate characteristics, educators are able to augment transformative learning strategies through tailored assignments and course activities.

## Limitations

Limitations in this study consisted of data collection methods and generalizability. The data collection followed a structured format, which did not allow for further exploration of prominent inductive themes or ideas. Furthermore, though content analysis was the most appropriate qualitative coding methodology for this study, it is limited in drawing relationality between salient themes, which may have been useful when critiquing an interdependent stage-based learning framework. This study may have benefited from data triangulation such as interviews or focus groups after the conclusion of the exchange to support richer responses that may have proven beneficial in identifying the subtle changes along the transformative learning cycle.

Regarding generalizability, this VE only evaluated the impact on American students at one public university, and did not evaluate the impact on Egyptian students in this paper. Future work could examine both sides of the partnership of the VE to determine if any change was reciprocal.

## Data Availability Statement

The datasets presented in this study can be found in online repositories. The names of the repository/repositories and accession number(s) can be found below: https://original-ufdc.uflib.ufl.edu/IR00011740/00001.

## Ethics Statement

The studies involving human participants were reviewed and approved by University of Florida Institutional Review Board (IRB#: IRB202003293). Written informed consent for participation was not required for this study in accordance with the national legislation and the institutional requirements.

## Author Contributions

SC contributed to preliminary discussions regarding research design and corresponding methodologies, conducted data analysis procedures, wrote the pedagogical theory, results, and discussion sections of this manuscript. SM conducted data analysis procedures and wrote the results and limitations sections of this manuscript. EW contributed to preliminary discussions regarding research design and corresponding methodologies, collected data, and wrote the introduction section of this manuscript. NS contributed to preliminary discussions regarding research design and corresponding methodologies, as well as training and supervising data analysis procedures. All authors engaged in editing the manuscript. All authors contributed to the article and approved the submitted version.

## Conflict of Interest

The authors declare that the research was conducted in the absence of any commercial or financial relationships that could be construed as a potential conflict of interest.

## Publisher's Note

All claims expressed in this article are solely those of the authors and do not necessarily represent those of their affiliated organizations, or those of the publisher, the editors and the reviewers. Any product that may be evaluated in this article, or claim that may be made by its manufacturer, is not guaranteed or endorsed by the publisher.
